# Double seronegative myasthenia gravis with antiphospholipid syndrome: a case report

**DOI:** 10.1186/1752-1947-8-2

**Published:** 2014-01-01

**Authors:** Diana Dan, Pierre-Alexandre Bart, Jan Novy, Thierry Kuntzer, Carole Clair

**Affiliations:** 1 Department of Rheumatology and Clinical Immunology, University Hospital Insel, 3010 Bern, Switzerland; 2 Service of Internal Medicine, University Hospital CHUV, Lausanne, Switzerland; 3 Service of Neurology, University Hospital CHUV, Lausanne, Switzerland; 4 Department of Ambulatory Care and Community Medicine, University of Lausanne, Lausanne, Switzerland

**Keywords:** Antibodies against acetylcholine receptor, Antibodies against muscle-specific receptor tyrosine-kinase, Antibodies against ryanodine receptor, Antibodies against titin, Antiphospholipid syndrome, Autoimmune disorders, Seronegative myasthenia gravis

## Abstract

**Introduction:**

Myasthenia gravis is an autoimmune disease characterized by fluctuating muscle weakness. It is often associated with other autoimmune disorders, such as thyroid disease, rheumatoid arthritis, systemic lupus erythematosus, and antiphospholipid syndrome. Many aspects of autoimmune diseases are not completely understood, particularly when they occur in association, which suggests a common pathogenetic mechanism.

**Case presentation:**

We report a case of a 42-year-old Caucasian woman with antiphospholipid syndrome, in whom myasthenia gravis developed years later. She tested negative for both antibodies against the acetylcholine receptor and against muscle-specific receptor tyrosine-kinase, but had typical decremental responses at the repetitive nerve stimulation testing, so that a generalized myasthenia gravis was diagnosed. Her thromboplastin time and activated partial thromboplastin time were high, anticardiolipin and anti-β2 glycoprotein-I antibodies were slightly elevated, as a manifestation of the antiphospholipid syndrome. She had a good clinical response when treated with a combination of pyridostigmine, prednisone and azathioprine.

**Conclusions:**

Many patients with myasthenia gravis test positive for a large variety of auto-antibodies, testifying of an immune dysregulation, and some display mild T-cell lymphopenia associated with hypergammaglobulinemia and B-cell hyper-reactivity. Both of these mechanisms could explain the occurrence of another autoimmune condition, such as antiphospholipid syndrome, but further studies are necessary to shed light on this matter.

Clinicians should be aware that patients with an autoimmune diagnosis such as antiphospholipid syndrome who develop signs and neurological symptoms suggestive of myasthenia gravis are at risk and should prompt an emergent evaluation by a specialist.

## Introduction

The manifestations of myasthenia gravis (MG) are muscle fatigability and weakness, caused by antibody-mediated reaction against the nicotinic acetylcholine receptor (AChR) at the neuromuscular junction [1,2]. Muscle-specific receptor tyrosine-kinase (MuSK) is a protein associated with the AChR, playing a role in its assembly [3]. The antibodies (Ab) against AChR are mostly of immunoglobulin G1 (IgG1) and IgG3 subclass; they induce a reduction in the number of available AChR molecules through cross-linking and accelerated degradation and through complement-mediated destruction of the postsynaptic membrane, resulting in a decrease of muscular response. Unlike AChR-Ab, MuSK-Ab are predominantly IgG4 and unable to activate complement, but they probably activate and internalize MuSK, which leads to a reduction and dispersal of AChR [4]. Results from several electrophysiological studies suggest that MuSK-Ab disturb both pre- and postsynaptic functions [5]. They inhibit muscle cell proliferation and downregulate the expression of AChR. Other antibodies seen in MG are antibodies against titin and ryanodine receptor. They bind to skeletal and heart muscle and are found in up to 95% of MG associated with thymoma and in 50% of late-onset MG. Also, antibodies against lipoprotein receptor-related protein 4 were recently identified, but their pathogenic role is not clear. Further structures of the neuromuscular junction, like agrin and ColQ (the collagen-tail subunit of acetylcholinesterase) seem to act as autoantigens in MG, but information about their pathogenic role is lacking [6]. The first clinical signs of MG are diplopia and eyelid ptosis, defining the ocular form of the disease. In generalized MG the weakness extends to bulbar muscles (dysarthria, dysphonia, chewing and swallowing difficulties), trunk (respiratory difficulties), and limbs.

The differential diagnosis includes, for the ocular form: stroke, multiple sclerosis, thyroid ophthalmopathy, meningitis and chronic progressive ophthalmoplegia; for the generalized form: Lambert–Eaton syndrome, congenital myasthenia, botulism, Guillain–Barré syndrome and amyotrophic lateral sclerosis.

Of the patients, 50 to 90% are AChR-Ab positive, with the highest rate of “seropositivity” in the generalized form [2]. MuSK-Ab are positive in 40% of the patients who are AChR-Ab-negative with generalized form, and usually negative in the ocular form. Patients who are AChR-Ab-negative but MuSK-Ab-positive have more predominantly bulbar involvement and more severe disease, with poorer response to therapy. This emphasizes the predictive value of specific antibodies analysis in patients with MG [7]. After 12 months, 15% of the patients who are initially seronegative for AChR-Ab will become seropositive as recently shown by Chan *et al*. [8]. The same authors found that among patients who are persistently seronegative, 38% are MuSK antibody-positive, and 43% are seropositive for miscellaneous, not muscle-related antibodies.

The thymus is involved in the pathogenesis of the disease, with abnormalities in 75% of cases, of which 85% are thymic hyperplasia and 15% thymoma [9]. A common feature of the seronegative MG for AChR-Ab is the low frequency of the thymic pathology [10,11].

MG may be associated with other autoimmune disorders such as thyroiditis, vitiligo, rheumatoid arthritis, connective tissue diseases or antiphospholipid syndrome (APS) [1].

APS is a disorder that manifests clinically as recurrent venous or arterial thrombosis and fetal loss. Laboratory abnormalities include evidence of a circulating anticoagulant or elevated levels of antibodies against membrane anionic phospholipids or their associated plasma proteins. Classification criteria are listed in Table 1[12].

**Table 1:** Revised classification criteria for the antiphospholipid syndrome

Antiphospholipid syndrome is present if at least one of the clinical criteria and one of the laboratory criteria that follow are met*	
CC1. Vascular thrombosis	One or more clinical episodes of arterial, venous, or small vessel thrombosis, in any tissue or organ**
CC2. Pregnancy morbidity	a) One or more unexplained deaths of a morphologically normal fetus at or beyond the 10^th^ week of gestation, with normal fetal morphology documented by ultrasound or by direct examination of the fetus, or
	b) One or more premature births of a morphologically normal neonate before the 34^th^ week of gestation because of: (i) eclampsia or severe preeclampsia, or (ii) recognized features of placental insufficiency, or
	c) Three or more unexplained consecutive spontaneous abortions before the 10^th^ week of gestation, with maternal anatomic or hormonal abnormalities and paternal and maternal chromosomal causes excluded
LC1. Lupus anticoagulant	present in plasma on two or more occasions at least 12 weeks apart
LC2. Anticardiolipin antibody	Of IgG and/or IgM isotype in serum or plasma present in medium or high titer (i.e. >40GPL or MPL, or > the 99^th^ percentile), on two or more occasions at least 12 weeks apart
LC3. Anti-β2 glycoprotein-I antibody	Of IgG and/or IgM isotype in serum or plasma (in titer > the 99^th^ percentile), on two or more occasions at least 12 weeks apart

*Abbreviations*: CC, clinical criteria; Ig, immunoglobulin; GPL, IgG phospholipid units; LC, laboratory criteria; MPL, IgM phospholipid units.

*Classification of antiphospholipid syndrome should be avoided if less than 12 weeks or more than 5 years separate the positive antiphospholipid test and the clinical manifestation.

**Thrombosis must be confirmed by objective validated criteria (that is, unequivocal findings of appropriate imaging studies or histopathology). For histopathologic confirmation, thrombosis should be present without significant evidence of inflammation in the vessel wall. Antiphospholipid syndrome categories: I: more than one laboratory criterion present (any combination); IIa: lupus anticoagulant present alone; IIb: anticardiolipin antibody present alone; IIc: anti-β2 glycoprotein-I antibody present alone.

APS occurs alone or in association with systemic lupus erythematosus or other rheumatic or autoimmune disorders. Not all people with the above-mentioned antibodies experience clinical complications, suggesting that other factors related to antibody specificity or host susceptibility may play a role.

## Case presentation

A 42-year-old Caucasian woman was admitted to our hospital because of fluctuating diplopia, ptosis, dysphagia, dysarthria and fatigable chewing at the end of meals or at the end of the day. Also, she would not answer the phone anymore because of her fear of not being able to finish a conversation. Her medical history included APS (that had led to three spontaneous abortions) and transitory hypothyroidism. She took acetylsalicylic acid 81mg daily.

On examination she had dysarthria and weakness of facial muscles, tongue and neck. She displayed progressive fatigability in upward gaze during the Simpson test. Repetitive ulnar nerve stimulation showed abnormal (>10%) decrement. The results of tests to detect antibodies against AChR, MuSK and titin were negative. A generalized MG was diagnosed. Her thoracic computed tomography scan was normal. Thromboplastin time and activated partial thromboplastin time were high, as an expression of the APS; anticardiolipin and anti-β2 glycoprotein-I antibodies were elevated. Laboratory findings are shown in Table 2.

**Table 2:** Laboratory findings in our patient

**Parameters**		**Units**	**Normal**
White blood cells	7.0	10^9^/L	4.0–10.0
Red blood cells	131	10^9^/L	117–157
Platelets	220	10^9^/L	150–350
Erythrocyte sedimentation rate	**32**	mm/h	<20
Thromboplastin time	**80**	%	85–125
Activated partial thromboplastin time	**37**	sec	21–33
Thyroid-stimulating hormone	2.95	mU/L	0.20–3.50
Myasthenia gravis antibodies			
Anti-titin	0.6	Quot.	<0.9
Anti-MuSK	0.01	nmol/L	<0.05
Anti-AChR	<0.02	pmol/L	<0.02
Rheumatoid factor	<11	UI/mL	<20
Antinuclear antibodies	1:320, Positive homogenous		<1:80
Anti-dsDNA antibodies	60	UI/mL	<200
Anti-nucleoproteins antibodies	9	U	<20
Antineutrophil cytoplasmic antibodies			
cANCA	Negative		
pANCA	Negative		
Anticardiolipin antibodies			
IgG, A, M (screening-test)	**2.0**		<1.0
IgG	**21.0**	GPL	<15
IgM	**27.0**	MPL	<12.5
Anti-β2 glycoprotein-I antibody			
IgG, A, M (screening-test)	**5.4**		<1.0
IgG	**48.0**	GPL	<20
IgM	**53.0**	MPL	<20

Pathologic values in bold.

*Abbreviations*: AChR, acetylcholine receptor; ANCA, antineutrophil cytoplasmic antibodies; dsDNA, double-stranded DNA; h, hour; GPL, IgG phospholipid units; Ig, immunoglobulin; MPL, IgM phospholipid units; MuSK, muscle-specific receptor tyrosine-kinase; Quot, Quotient; sec, seconds.

She had a good clinical response when treated with pyridostigmine, prednisone and azathioprine.

## Discussion

A review of the literature suggests that there could be a link between APS and MG, as both are autoimmune disorders. Exceptional case reports with this particular clinical association are listed in Table 3.

**Table 3:** Cases with myasthenia gravis associated with antiphospholipid syndrome

**Case**	**Manifestations of**	**Manifestations of**	**Laboratory findings**
	**myasthenia gravis**	**antiphospholipid syndrome**	
Kaji *et al.*[13]	Woman, 47	Began after thymectomy	Prolonged aPTT, thrombocytopenia, anti-β2 glycoprotein-I antibody positive
	Diplopia, weakness of arms and legs with prolonged exertion	Phlebothrombosis of the lower extremity, multiple strokes	Anti AChR-Ab 61nmol/L (N <0.2nmol/L)
Shoenfeld *et al.*[14]	Woman, 32	Began after thymectomy Abortion, stroke and spleen artery thrombosis	Anticardiolipin antibodies positive
	Hand weakness		Anti AChR-Ab positive
Watanabe *et al.*[15]	Woman, 40 Diplopia, bilateral ptosis	Abortion, pulmonary embolism	Prolonged aPTT, thrombocytopenia, anti-β2 glycoprotein-I antibody and lupus anticoagulant positive
			Anti AChR-Ab 490nmol/L (N <0.2nmol/L)
Present case	Woman, 42 Fluctuating faciobulbar weakness	Abortions	Prolonged aPTT, anti-β2 glycoprotein-I and anticardiolipin antibody positive
			Antinuclear-Ab positive
			Anti AChR-Ab, MuSK-Ab, titin-Ab negative

*Abbreviations*: Ab, antibodies; AChR, acetylcholine receptor; APTT, activated partial thromboplastin time; MuSK, muscle-specific receptor tyrosine-kinase.

In two cases, APS became manifest years after a thymectomy. Patients with MG who have been thymectomized display over the years mild T-cell lymphopenia, hypergammaglobulinemia and B-cell hyper-reactivity, most probably related to the loss of suppressor T-cells. This could lead to an associated autoimmune disease. However, this explanation does not apply either for the third or for our own case, APS having been diagnosed years before MG.

Equally, many patients with MG test positive for a large variety of autoantibodies, even in the absence of thymectomy. In a serologic study, Sanmarco and Bernard [16] found in 71 patients with MG a prevalence of 25% of IgG-class anticardiolipin antibodies, and these were significantly associated with thymic abnormalities such as thymoma or thymic hyperplasia. This mechanism could favor the emergence of other autoimmune diseases, such as APS.

## Conclusions

We have reported a case of MG in which an APS was diagnosed a few years before. The patient tested negative for both AChR-Ab and MuSK-Ab, which is a very rare situation. Recently published data have shown that MuSK-positive and MuSK-negative MG are distinct clinical entities, with a better prognosis for the AChR-positive and MuSK-negative forms of the disease. This emphasizes the predictive value of specific antibodies analysis in patients with MG.

Very few other cases of concomitant MG and APS have been described and this raises the hypothesis of a continuum in certain autoimmune diseases. Little is known about the causality and the pathogenicity of these associated disorders. Contrary to our case, there might be a relationship between thymectomy in MG and occurrence of other autoimmune diseases such as APS but data are scarce and subject to controversy. It is expected that other mechanisms should be considered for explaining what could trigger the development of concomitant dysimmune response to different targets and this could probably be studied by chromatin immunoprecipitation assay, a powerful tool to study protein–deoxyribonucleic acid (DNA) interactions from different tissues.

Clinicians should be aware that patients with an autoimmune diagnosis such as APS who develop signs and neurological symptoms suggestive of MG are at risk and should prompt an emergent evaluation by a specialist.

## Consent

Written informed consent was obtained from the patient for publication of this manuscript. A copy of the written consent is available for review by the Editor-in-Chief of this journal.

## Abbreviations

Ab: Antibodies; AChR: Acetylcholine receptor; APS: Antiphospholipid syndrome; IgG: Immunoglobulin G; MG: Myasthenia gravis; MuSK: Muscle-specific receptor tyrosine-kinase.

## Competing interests

Diana Dan, Pierre-Alexandre Bart, Jan Novy, Thierry Kuntzer and Carole Clair do not report any conflict of interest.

## Authors’ contributions

DD examined the patient, collected the data, made the literature review and was a major contributor in writing. PAB helped with drafting the manuscript especially regarding the antiphospholipid syndrome. JN helped with drafting the manuscript and compiled data on myasthenia gravis. TK helped with drafting and reviewing the manuscript. CC helped with drafting and reviewing the manuscript. All authors read and approved the final manuscript.
